# Clinical Applications of Pressure-Volume Assessment in Congenital Heart Disease

**DOI:** 10.1016/j.jscai.2023.100599

**Published:** 2023-02-22

**Authors:** Gurumurthy Hiremath, Sarosh Batlivala, Ryan Callahan, Nikhil Thatte, Toby Rockefeller, Hythem Nawaytou, Surendranath V. Reddy, Tarique Hussain, Radomir Chabiniok, Ryan Butts, Joseph Vettukattil, E. Oliver Aregullin, Nael Aldweib, Daniel Burkhoff, Michael I. Brener

**Affiliations:** a Division of Pediatric Cardiology, Department of Pediatrics, Masonic Children’s Hospital, University of Minnesota, Minneapolis, Minnesota; b Division of Pediatric Cardiology, The Heart Institute, Cincinnati Children’s Hospital Medical Center, University of Cincinnati College of Medicine, Cincinnati, Ohio; c Department of Pediatrics, Children’s Hospital of Philadelphia, University of Pennsylvania, Philadelphia, Pennsylvania; d Department of Cardiology, Boston Children’s Hospital, Harvard Medical School, Boston, Massachusetts; e Interventional Pediatric Cardiology, University of Missouri-Kansas City School of Medicine, Children’s Mercy, Kansas City, Missouri; f Department of Pediatrics, UCSF Benioff Children’s Hospital and the University of California, San Francisco, California; g Pediatric Cardiology, Children’s Medical Center, Dallas, Texas; h Congenital Heart Center, Spectrum Health Helen DeVos Children’s Hospital, Grand Rapids, Michigan; i Division of Cardiovascular Medicine, Oregon Health Sciences University, Portland, Oregon; j Division of Cardiology, Columbia University Irving Medical Center/NewYork-Presbyterian Hospital, New York, New York

**Keywords:** pressure-volume loops, ventricular mechanics, congenital heart disease, single-ventricle physiology, pulmonary hypertension, myocardial performance

## Abstract

Ventricular pressure-volume (PV) loops offer unique insights into cardiovascular mechanics. PV loops can be instrumental in improving our understanding of various congenital heart diseases, including single ventricular physiology, heart failure, and pulmonary hypertension, as well as guiding therapeutic interventions. This review focuses on the theoretical and practical foundations for the acquisition and interpretation of PV loops in congenital heart disease and discusses their clinical applications.

## Highlights

• Pressure-volume loops are the gold standard for assessing myocardial performance.
• Pressure-volume loops have several applications in congenital heart disease.
• Modelled pressure-volume loops from advanced imaging can supplement invasive loops.

## Central Illustration

**Figure:**
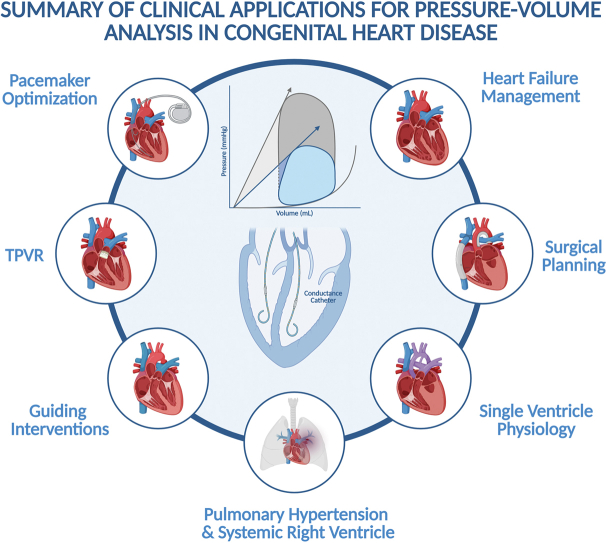
Summary of the various clinical applications of pressure-volume loop analysis in congenital heart disease. TPVR, Transcatheter pulmonary valve replacement.

## Introduction

Children with congenital heart disease (CHD) are a diverse population with significant hemodynamic derangements that often persist long after early reparative procedures. Residual lesions leading to abnormal loading conditions and prosthetic material commonly placed in the circulatory system adversely affect myocardial performance. In addition, patients with CHD undergoing multiple surgeries and those in whom the right ventricle (RV) supports systemic circulation are at especially higher risk for myocardial dysfunction.

Intracardiac pressure-volume (PV) loops offer the ability to elucidate these complex ventricular mechanics and hemodynamic interactions in ways traditional testing cannot.^1^^,^^2^ Noninvasive assessment using echocardiography and cardiac magnetic resonance (CMR) can provide some of the information yielded by PV analysis.^3^, ^4^, ^5^, ^6^, ^7^ There are limitations to these techniques, including their variability under different loading conditions and their focus on chamber volume and morphology. Intracardiac PV loops address these shortcomings by combining simultaneous measurements of pressure and volume to generate load-independent measures of systolic and diastolic function. Therefore, it is considered the ‘gold standard’ method for evaluating myocardial performance. Herein, we review the basic techniques and principles of PV analysis as well as appropriate clinical and research-related applications in CHD (Central Illustration).

**Central Illustration:**
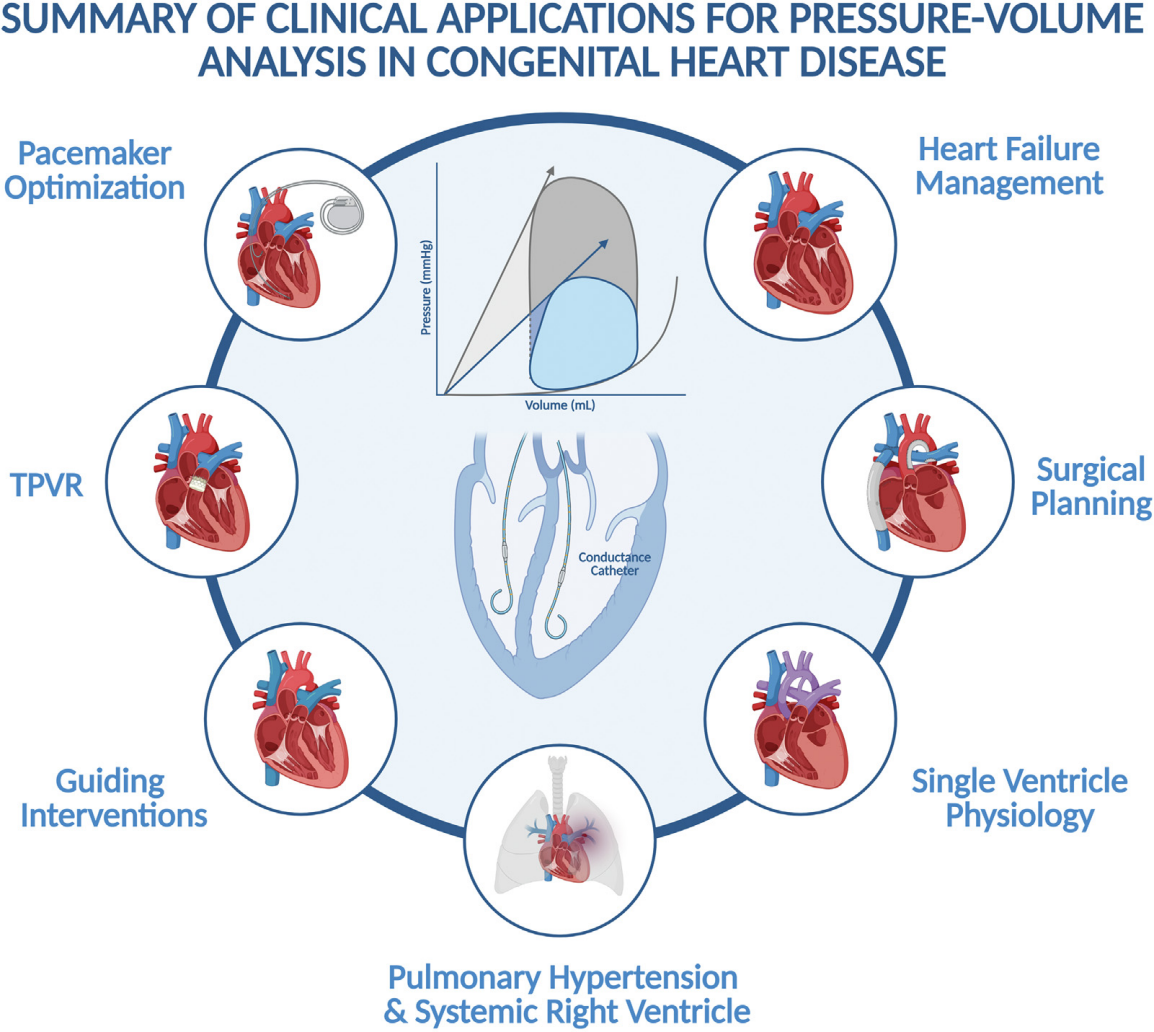
Summary of the various clinical applications of pressure-volume loop analysis in congenital heart disease. TPVR, Transcatheter pulmonary valve replacement.

## Theoretical foundations for PV loop analysis and interpretation

**Figure 1:**
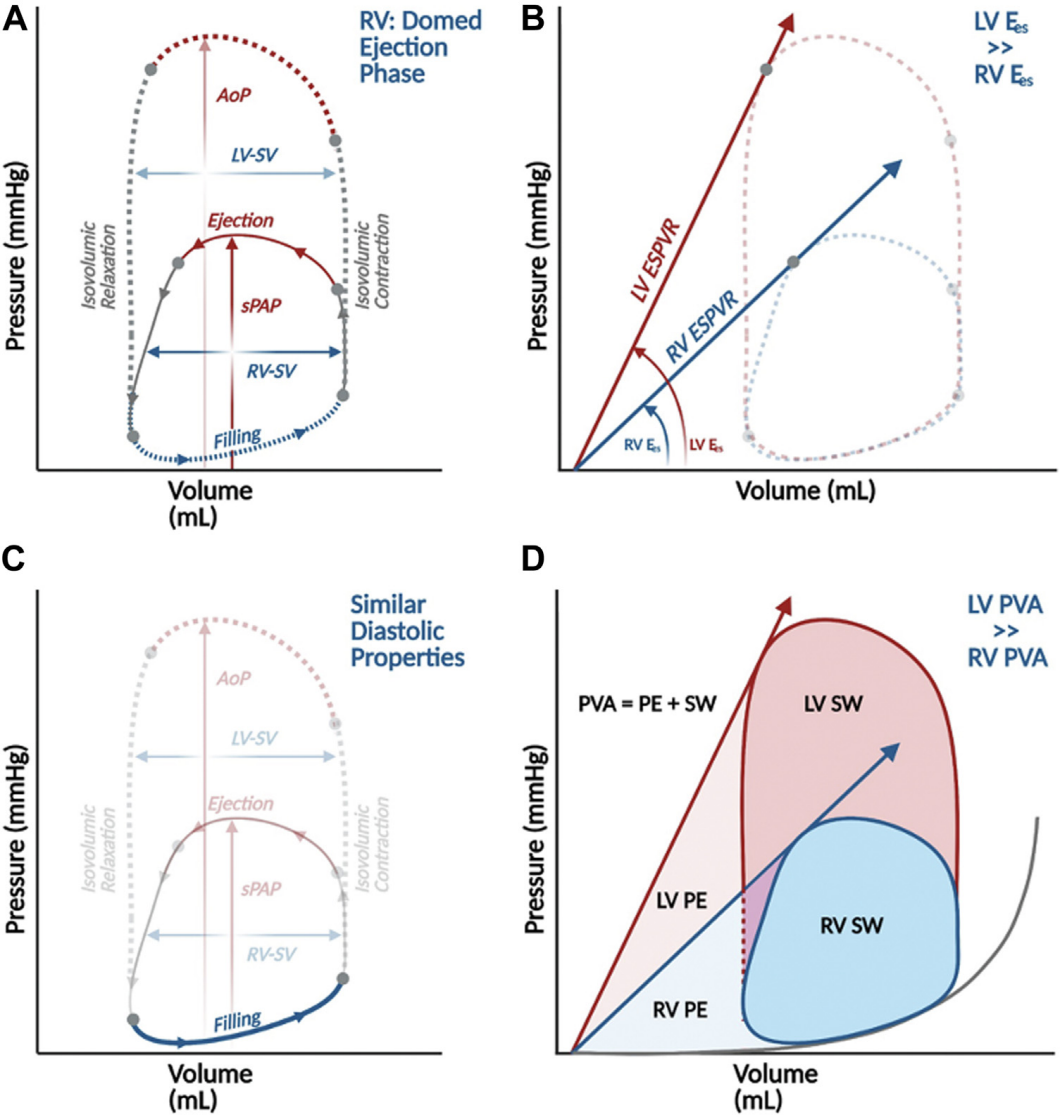
**Theoretical considerations for PV loops analysis and interpretation**. Examples of RV and LV PV loops superimposed on each other. (A) AoP, aortic systolic pressure; sPAP, systolic pulmonary artery pressure; LV, left ventricle; RV, right ventricle; SV, stroke volume; (B, C) E_a_, effective arterial elastance; EDPVR, end-diastolic pressure-volume relationship; E_es_, end-systolic elastance; ESPVR, end-systolic pressure-volume relationship; (D) PE, potential energy; SW, stroke work; PVA, Pressure-volume area.

Conceptually, PV loops describe ventricular hemodynamics during one cardiac cycle (Figure 1A). The 4 sides of the loop denote the cardiac cycle’s 4 phases: beginning from the bottom right corner, (1) isovolumic contraction, (2) ejection, (3) isovolumic relaxation, and (4) filling. The width of the loop represents the stroke volume (SV), while the height of the loop represents the peak ventricular pressure. In the left ventricle (LV), this is equivalent to peak arterial systolic blood pressure (assuming no transvalvular stenosis), and in the RV, it is equivalent to peak pulmonary artery (PA) systolic pressure. LV PV loops are more rectangular and have clearly defined isovolumic contraction and relaxation phases; in contrast, in the normal RV, PV loops have a more domed systolic ejection phase, and there is often no clear isovolumetric relaxation phase, such that systolic pressure often decays to diastolic pressure by the end of systole. The area inside the loop is the stroke work (SW) (Figure 1D).

The PV loop dimensions are defined by 2 fundamental relationships: the end-systolic PV relationship (ESPVR) and the end-diastolic PV relationship (EDPVR). The ESPVR summarizes ventricular systolic function and is reasonably linear, with slope E_es_ (end-systolic elastance) and volume axis intercept V_0_ such that ESP = E_es_ (ESV-V_0_) (Figure 1B; ESP = end-systolic pressure, ESV = end-systolic volume). V_0_ represents the unstressed volume, which is the volume required to fill the ventricle before pressure rises. Shifts of the ESPVR occur with changes in ventricular *contractility,* such that increases in contractility are associated with upward/leftward shifts of the ESPVR (Figure 1B). The RV ESPVR is roughly one-fifth as steep as the LV ESPVR, considering normal peak pulmonary pressures are about a fifth of peak systemic pressures.

The EDPVR (Figure 1C) is constructed by connecting the end-diastolic PV points of each loop and reflects the extent of relaxation and the passive ventricular properties when all actin-myosin bonds are uncoupled. It is nonlinear, with a shallow slope at lower ventricular volumes and a steeper slope at higher ventricular volume ranges. EDPVRs generally span the same and relatively limited range of pressures, from 0 mm Hg to a maximum of 30 or 40 mm Hg even among different species, even while the volume required varies to achieve this same range of pressures. While EDPVR is easily measured ex vivo, in vivo assessment of the EDPVR is challenging due to the need to obtain PV loops over a range of volumes using preload modification techniques. To overcome this, single-beat estimates of EDPVR were developed and validated.^8^ Simplified equations such as P = β (e^α (V-V0)^-1) or P = βV^α^ provide curved fits for EDPVR over physiological ranges where constants like α and β relate to mechanical properties of the ventricle, ventricular geometry, the myocardium, and extracellular matrix.^9^ Changes in myocardial properties (eg, fibrosis, ischemia, edema), physiological or pathological remodeling can all cause shifts in EDPVR. A stiffer, less compliant ventricle is illustrated on the PV diagram by a steeper EDPVR slope that is shifted upwards and to the left.^10^ In contrast to the ESPVR, the RV and LV have similar EDPVRs because their chamber volumes are similar.^11^

Several fundamental parameters that influence ventricular performance are also readily depicted on the PV diagram. Ventricular *preload*, for example, can be related to end-diastolic pressure (EDP) or end-diastolic pressure volume (EDV). Similarly, ventricular *afterload* is depicted on the PV diagram by the “effective arterial elastance” (E_a_) line. The E_a_ line connects the end-systolic pressure and volume coordinates (ESV, ESP) and a point at EDV. In the LV, this is often simplified to the EDV volume axis intercept (ie, EDV, 0), because downstream pressure in the systemic circuit (ie, central venous pressure) is negligible when compared with systolic arterial pressure. In contrast, pulmonary capillary wedge pressure may represent a large fraction of PA systolic pressure.^12^ As a result, E_a_ may be better captured in the RV by connecting the end-systolic point with the (EDV, pulmonary capillary wedge pressure) coordinate. As an extension of this concept, the degree to which systolic function is matched to afterload can also be described on the PV diagram by the ratio of end-systolic elastance to effective arterial elastance (E_es_/E_a_); this ratio is the foundation of the concept of ventricular-vascular interaction, or ventricular-arterial (VA) coupling.

In addition to providing a platform for explaining ventricular mechanics, the PV diagram also provides a construct for understanding the determinants of myocardial oxygen consumption. Myocardial oxygen consumption per beat is linearly related to the ventricular PV area (PVA), which is the sum of the SW and the potential energy. The potential energy is the area bounded by the ESPVR, the EDPVR, and the diastolic portion of the PV loop and represents the residual energy stored in the myofilaments at the end of systole that was not converted to external work (Figure 1D).

## Practical considerations for performing invasive PV loops in children

### Infrastructure

**Figure 2:**
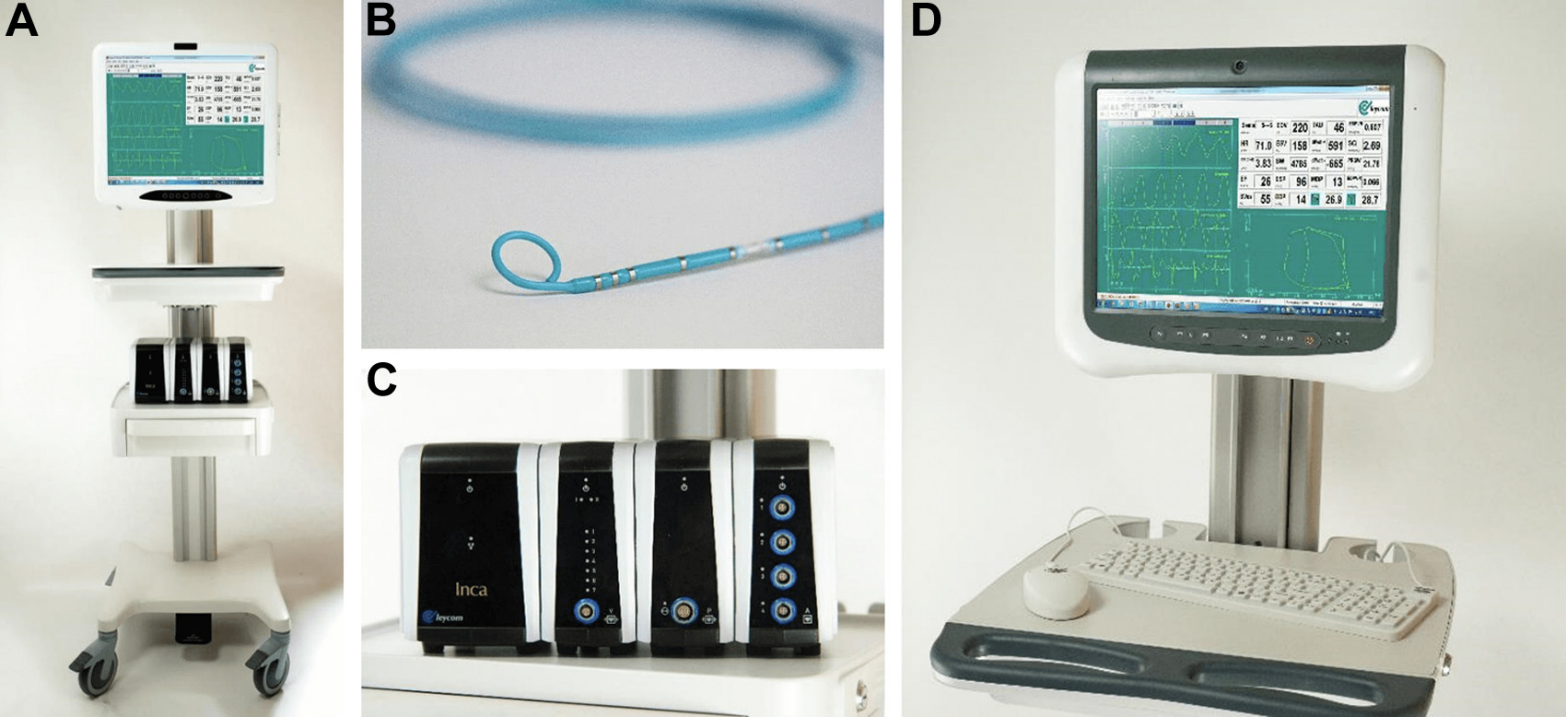
**Infrastructure needed for invasive PV loop assessment**. CD Leycom’s Inca PV Loop System (A, C) CD Leycom high-fidelity conductance catheter with tip electrodes and pressure sensor (B). Conduct NT software and monitor (D). PV, pressure-volume.

Currently, CD Leycom is the only manufacturer and distributor of the equipment needed for invasive PV loop analysis in humans. This includes the high-fidelity conductance catheters, connection cables, and the Inca (INtraCardiac Analyzer) PV Loop System with the Conduct NT software (Figure 2). The high-fidelity conductance catheters come in 2 sizes: a 4F catheter version without a lumen and a 7F catheter version with a 0.025-inch lumen. Pressure is measured in real time using a solid-state high-fidelity pressure sensor located in the middle of an array of 12 equally spaced electrodes at its tip. Volume is estimated by measuring voltage drops between successive pairs of electrodes, which are proportional to the cross-sectional area of the ventricular blood pool. Total chamber volume is obtained as a summation of the segmental chamber areas after excluding segments that are outside the ventricle, which can be identified in real time by segmental PV loops that are not proceeding in a counterclockwise fashion during the cardiac cycle (ie, losing volume during systole). Additional aspects of the conductance method that are critical for understanding how these complex signals are processed and translated into PV loops have been previously described.^1^^,^^2^^,^^13^^,^^14^ The conductance catheters are connected to the Inca PV Loop System to obtain real-time PV loops. Further post-processing and analysis of the data can be performed using Conduct NT or other similar software.

### Catheter delivery and positioning in children

For LV PV loops, placement of the catheter with its tip in the ventricular apex can be accomplished using a retrograde transaortic approach via an arterial access or an antegrade transseptal approach via a venous access. Due to the stiffness of the conductance catheter, using a standard pigtail catheter to reach the LV apex followed by an exchange for the 7F conductance catheter over a stiff guidewire (such as the 0.025-inch Amplatz extra-stiff wire or a 0.018-inch Platinum-Plus wire [Boston Scientific]) is preferred. Such an exchange is not possible with the 4F lumenless conductance catheter. In smaller children where sheath size is a concern, the use of a long sheath to traverse the curve of the aortic arch aids in positioning the 4F catheter into the LV apex. For RV PV loops, the conductance catheter is placed with the tip at the RV apex. This can be most easily achieved through an internal jugular vein approach. A pre-shaped sheath (such as the Mullins transeptal sheath, Cook Medical) with a gentle curve helps in guiding the catheter to the RV apex. A femoral venous approach may be more challenging, especially if using the 4F catheter, in which case the use of a pre-shaped or steerable long sheath may help. We recommend using a sheath that is at least one French size larger than the conductance catheter so the side port can be used for infusions of hypertonic saline, which is needed for volume calibration. Proper catheter positioning is confirmed by the presence of appropriately shaped segmental PV loops (trapezoidal shape, counterclockwise movement of PV points during the cardiac cycle). In smaller children, it is common for some segments to be outside the ventricle, resulting in “clockwise” or “Figure of 8” loops that need to be excluded from the analysis. Once in a proper position, it is recommended to secure the catheter to avoid recalibration with catheter movement.

### Pressure and volume calibration

Automatic pressure calibration is performed after the high-fidelity pressure sensor is emersed for 15 seconds in saline-soaked gauze. Volume calibration is performed after inserting the catheter into the ventricle or during post-processing. There are 2 basic methods for volume calibration both with inherent limitations^1^^,^^14^, ^15^, ^16^: (1) using hypertonic saline or (2) using cross-sectional imaging such as CMR, computerized tomography, or 3D echocardiography. The principles of volume calibration using hypertonic saline have been previously described.^14^^,^^16^ This requires a 5 to 10 mL infusion of hypertonic saline (5%-10%) over 2 to 3 seconds upstream from the conductance catheter (through the side port of the sheath if doing RV PV loops or through a right heart catheter if doing LV PV loops). This alters the blood pool’s conductance and provides the value for the offset factor (V_p_, called the parallel conductance offset) that is needed for converting the conductance signal, C (*t*), to absolute volumes. Thermodilution or modified Fick estimates of cardiac output are performed to obtain the SV, which is then used to calculate a gain factor (α) using the formula: SV = α ▪ C (*t*). The hypertonic saline method has the advantage of being able to be performed immediately at the time of the procedure. This method, however, has several methodological and theoretical limitations intrinsic to the conductance principle,^14^ including the assumption that the equipotential electrical field lines in the ventricular are all parallel and the negligible conductance of the structures around the ventricular chambers. There are some technical limitations of hypertonic saline infusion, such as the wide variance of a linear regression line created from a small number of points during the change in ventricular volumes. The limitation of thermodilution cardiac output, which may be accentuated in valvar insufficiency and intracardiac shunts, also affects accuracy. The error in the volume estimation by the parallel conductance method may be as high as 8% to 15%.^17^^,^^18^

The administration of hypertonic saline may also be of some concern in small children and in those with renal impairment or heart failure (HF). With the small volumes used, the amount of sodium administered is low and carries a low risk. An alternate approach to volume calibration utilizes end-diastolic and end-systolic volumes obtained by imaging, typically by CMR or 3D echocardiography performed in close proximity to the invasive PV loop analysis. Echocardiographic volume calibration, similar to the hypertonic saline method, can be performed at the time of the procedure but is limited by the ability to acquire reliable images to estimate volumes, especially of the RV, and repeated imaging with changing loading conditions can be challenging. CMR, which provides the most reliable reference volumes, typically cannot be obtained simultaneously at the time of the procedure in most centers, and hemodynamic alterations occurring between the time of CMR acquisition and procedure may influence volumes. Repeated volume calibrations may be needed in cases of catheter displacement or changes in hemoglobin.

### Preload reduction techniques

**Figure 3:**
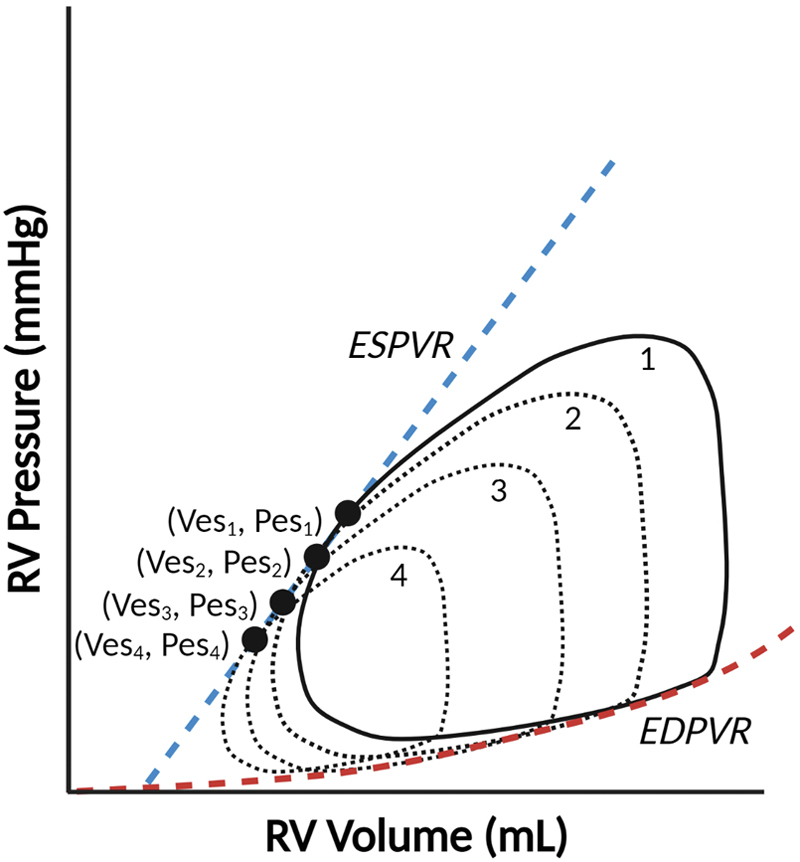
**Preload modification by vena caval occlusion helps determine the end-systolic and end-diastolic relationships by linear regression**. EDV and SV decrease from loop 1 to loop 4 in this theoretical example of RV PV loops. EDPVR, end-diastolic pressure–volume relationship; ESPVR, end-systolic pressure–volume relationship; V_es_, end-systolic volume; P_es_, end-systolic pressure. EDV, end-diastolic volume; PV, pressure-volume; RV, right ventricle; SV, stroke volume.

The classic measurements of ESPVR and EDPVR (Figure 3) require transient modulations of loading conditions. The preferred method involves balloon occlusion of the inferior vena cava, but this warrants additional femoral or jugular venous vascular access and may not be feasible depending on anatomic considerations.^13^ Other methods of recapitulating reduced inferior vena cava flow include the Valsalva maneuver (which may be easily achieved, especially in intubated children) or external abdominal compression. However, each of these methods may independently affect sympathetic tone, ventricular afterload, and hence ventricular contractility. In the absence of dysautonomia, sympathetic tone increases during phase II of the Valsalva maneuver, thereby artificially increasing the ventricular contractility and altering the ESPVR curve.^19^ To overcome these limitations, single beat estimations of ESPVR and EDPVR have been developed, validated, and previously described.^1^^,^^8^^,^^20^

## Invasive PV loops in validation of noninvasive assessments of ventricular function in CHD

Several studies have used PV loop indices of ventricular systolic and diastolic function to validate echocardiographic measures of cardiac function in children (Table 1). Chowdhury et al^3^^,^^4^ published 2 investigations on the comparison of echocardiographically derived measurements of LV function with those obtained from PV loop analysis in 24 children with structurally normal hearts. Butts et al^7^ compared echocardiographic measures of ventricular systolic function with invasive PV loop indices in 15 patients with single-ventricle physiology, with heterogeneous and variable ventricular dominance and stages of palliation. Fractional area change (FAC) had a mild correlation with E_es_ (*r* = 0.69, *P* < .01), dP/dt max (*r* = 0.59, *P* = .03), and SW/EDV (*r* = 0.64, *P* = .01), but there was no significant correlation with other echocardiographic measurements.

**Table 1:** Correlation of invasive PV loop indices with noninvasive assessments in children

Reference, year	Imaging modality	No. of patients	Age, y (median, range)	Cardiac anatomy	Key findings
Butts et al,^6^ 2015	Echo	15	3.8 (0.3-19.3)	Single-ventricles variable morphology and surgical palliation (3 shunt, 5 Glenn, 7 Fontan)	• FAC correlated with E_es_ (*r* = 0.69, *P* < .01), SW/EDV (*r* = 0.64, *P* = .01), and dP/dt max (*r* = 0.59, *P* = .03) • Long SF correlated with dP/dt max (*r* = 0.61, *P* = .02) • MPI, AV valve dP/dt, and isovolumic acceleration did not correlate with pressure–volume loop indices.
Steflik et al,^21^ 2017	Echo	17	8 (5-17)	Single ventricles with Fontan (12 right dominant, 5 left dominant)	• RV-dominant morphology: Circumferential strain (r = -.72, *P* ≤ .01) and strain rate (*r* = -.61, *P* = .04) correlated with E_a_/E_es_ • LV-dominant morphology: Longitudinal strain rate correlated with E_es_ (*r* = -.98, *P* ≤ .01). Longitudinal EDSR correlated with tau (*r* = -.90, *P* = .04)
Chowdhury et al,^3^ 2016	Echo	24	9.1 ± 5.6 (mean, SD)	Normal anatomy (18 transplant, 6 small PDA or CAF)	• Speckle-tracking derived longitudinal strain is associated ICCI (β = -0.54, *P* = 0.02) • Invasive E_es_ correlated best with echocardiographic Ees by the method of Tanoue (*r* = 0.85, *P* < .01)

AV, atrioventricular; CAF, coronary artery fistula; dP/dt, derivative of pressure over time; E_a_, effective arterial elastance; E_es_, end-systolic elastance; EDSR, early diastolic strain rate; EDV, end-diastolic volume; FAC, fractional area change; ICCI, invasive composite contractility index; LV, left ventricle; MPI, myocardial perfusion imaging; PDA, patent ductus arteriosus, PV, pressure-volume; RV, right ventricle; SD, standard deviation; SF, shortening fraction; SW, stroke work.

Steflik et al^21^ found that ventricular morphology influenced the correlation between invasive and noninvasive functional parameters. In patients with a right dominant morphology, circumferential measures of systolic deformation correlated best with PV loop measures of ventricular-vascular coupling, whereas in patients with a left dominant morphology, longitudinal measures of deformation correlated best with PV loop systolic indices (Table 1).

## PV loops obtained using interventional CMR imaging

Interventional CMR (ICMR) imaging is a novel technique where cardiac catheterization is done with CMR guidance.^22^ While pressures inside the ventricle are recorded with a fluid-filled catheter, the ventricular volumes can be accurately obtained by CMR by processing cine MR image data.^23^, ^24^, ^25^ Volumetric and flow data can be combined with simultaneous hemodynamic data to generate PV loop derived indices of ventricular function.^26^ This can be achieved sequentially (ie, CMR volumes are recorded first and then pressure measurements in the catheterization laboratory, or vice versa) or simultaneously if an ICMR suite is available. While sequential analysis can be performed in most centers, its main disadvantage is that the volume and pressure datasets are not obtained exactly at the same time, lending to potential errors due to different loading conditions and varying heart rates. The latter, ICMR-based PV loop analysis requires a dedicated imaging and procedural suite, which is not available ubiquitously. Wong et al^26^ used this technique to acquire comprehensive hemodynamic and ventricular function data without the use of ionizing radiation in multiple conditions (ie, rest, dobutamine) in 10 children with mean age of 8.6 years (3.5-11.6 years) with hypoplastic left heart syndrome status post-Fontan palliation and with exercise intolerance. Magnetic resonance-compatible catheters were placed in the systemic RV and branch pulmonary arteries to record pressures at rest, with dobutamine infusion at 10 and 20 μg/kg/min. Cine short-axis stacks of the ventricle were performed at each condition and used to construct PV loops. The findings highlighted the failure to augment preload as the primary limitation to cardiac output augmentation in this subgroup.

In comparison to the PV loops assessed by conductance catheters, ICMR-based pressure and volume signals, even though obtained simultaneously, are not perfectly synchronized for several reasons. The time offset between the catheter-derived pressure data and the CMR image (and thus the volume data) depends on several factors, such as the type and settings of the CMR sequence, the duration and shape of the QRS complex, or the type of catheter. Gusseva et al^27^^,^^28^ proposed using a biophysical heart model to synchronize the LV pressure and volume datasets. The proposed time-synchronization strategy using patient-specific biophysical modeling can substitute for a missing part of the cardiac cycle in the volume waveform.^27^

## Clinical applications of PV loops in CHD

### PV loop applications for assessing single-ventricle physiology

Despite advances in therapy, children with single-ventricle physiology exhibit significant hemodynamic limitations, exercise intolerance, and decreased long-term survival. Redington et al^29^ demonstrated the PV loop characteristics of the morphologic RV in children with single ventricles in the early 1990s. When exposed to systemic pressures, the RV becomes very similar to that of a morphologic LV. Butts et al^7^ later attempted to understand the differences in the PV relationship between a single RV with a Sano shunt and a single LV with a Blalock-Thomas-Taussig shunt. While demonstrating the feasibility of PV loops in smaller children, they observed the near elimination of isovolumic contraction, leading to a decreased SW in the single RV population. This finding was likely due to the different filling characteristics and volume loading than to the different ventricular dominance, as the preload in these groups of patients was significantly different (Qp/Qs of 0.54 vs 1.15). The importance of preload in defining the cardiovascular performance in single ventricles was highlighted in the study of 10 Fontan patients undergoing dobutamine stress testing.^26^ The cardiac index increased with low-dose dobutamine (10 μg/kg/min), but not with higher-dose dobutamine (20 μg/kg/min), despite a slight increase in heart rate (*P* = .002). A fall in SV occurred (*P* = .014) despite an increase in contractility (74% increase, *P* = .045) and a well-coupled ventriculo-arterial elastance ratio. End-diastolic pressure and early active relaxation, markers of diastolic function, were also normal at rest, while pulmonary vascular resistance (PVR) remained low throughout the experiment. Preload was maintained at low doses of dobutamine but fell at the high dose (*P* < .008), leading to the conclusion that the ceiling of maximal flow through the Fontan circuit could be the primary physiologic limitation in these patients. On the other hand, Schlangen et al,^30^ in a study of 56 children with hypoplastic left heart syndrome at a median age of 2.6 years after their Fontan completion, identified elevated arterial elastance as the main hemodynamic derangement in this population.

Although high resting PVR is often blamed for Fontan failure, there are several interrelated and complex hemodynamic changes such as endothelial dysfunction, pulmonary vascular remodeling, post-capillary pulmonary hypertension, abnormal ventricular diastolic function, residual valvar incompetence, and collateral vessels causing volume overload that contribute to it.^31^^,^^32^ The least understood factors are impairment in ventricular diastolic properties and compliance; the concept of HF with preserved ejection fraction (HFpEF) is yet to be defined in this population. Diastolic dysfunction in single ventricles could be a result of several factors, including dyssynchrony, hypoxia, geometry, scarring or fibrosis, longstanding volume overload before Fontan palliation, and subsequent decreased ventricular preload.^31^ Volume challenge has been used to uncover diastolic dysfunction, but it is nonphysiological and difficult to interpret because most patients have altered venous capacitance, veno-venous collaterals, and intrapulmonary shunting. There is renewed interest in the use of invasive hemodynamics during exercise in this population. Miranda et al^33^ compared exercise hemodynamics between 24 adults post-Fontan and 48 patients with HFpEF and noncardiac dyspnea and observed markedly abnormal single-ventricle compliance in this population despite lower resting and exercise pulmonary artery wedge pressure. The same group had previously reported that an abnormal slope of increase in mean pulmonary pressure relative to cardiac output exacerbated during exercise could be superior to a resting PVR in identifying pulmonary vascular limitations in Fontan physiology.^32^ These studies suggest exercise invasive hemodynamics may represent a novel tool for the diagnosis of diastolic dysfunction in Fontan patients. Differentiating and unmasking the components of single ventricular diastolic failure in the setting of preload deprivation and the absence of increased afterload may be possible by performing PV loops, possibly with exercise challenge, as suggested by Budts et al^34^ using modeled PV loops in this population. This may provide a detailed quantification of ventricular and pulmonary vascular mechanics and allow for an incremental understanding of the extent of single-ventricle HF compared to the sole volumetric assessment with ejection fraction (EF) values that is currently in use. The change in filling pressure with exercise in compliant vs noncompliant ventricles may help decide what form of afterload reduction is appropriate for a given patient. It may also help decide when to close and when to create a Fontan fenestration, especially in patients with protein losing enteropathy and normal PA pressures at rest.

Arterial elastance is also abnormal in a subset of patients with a single ventricle with underlying aortic arch abnormalities and negatively impacts systolic ventricular function early in their staged palliation. However, the increased arterial elastance is also associated with ventricular diastolic stiffness; the resultant long-term effect on the Fontan circulation will need further studies with PV loops. Septatability in children with borderline ventricles is another area where PV loops can play an important role. Currently, this is assessed based on the morphologic and anatomic substrates without regard to functional morphology, diastolic function, or reserve.

### Characterization of the systemic RV using RV PV loops

**Figure 4:**
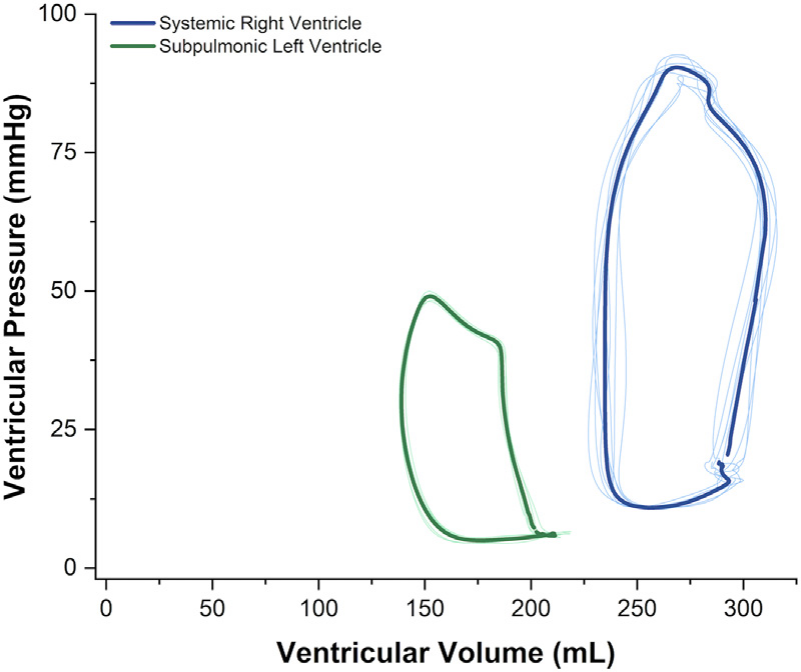
**PV relationship of the morphologically RV in systemic position (blue), and of the morphologically LV in pulmonary position (green) in a patient with L-loop transposition of great arteries using conductance catheters**. The systemic RV has a prominent isovolumic contraction and relaxation phases and more closely resembles typical LV loops. LV, left ventricle; PV, pressure-volume; RV, right ventricle.

A systemic RV is encountered in approximately 10% of CHD and remains poorly understood with a guarded prognosis.^35^ Currently, recommendations capitalize on echocardiography and CMR to measure RV size and EF as a surrogate for RV contractility.^36^^,^^37^ However, a systemic RV has a different phenotype and adaptations than a subpulmonic RV, which is governed by higher resistance and a less compliant aorta. While systemic and subpulmonic RVs have a similar myocardial architecture consisting of a superficial circumferential layer and a deep longitudinal layer, they conspicuously lack a middle layer of circular fibers that generally make up more than half the wall thickness of a morphological LV.^38^^,^^39^ In addition, septal contraction contributes 20% to 40% to subpulmonic RV SV, which may not be the case for a systemic RV.^36^^,^^40^ Hence, RV contractility and systolic function may not be accurately assessed by RV size or EF in individuals with systemic RVs. These differences in adaptation and mechanics have been demonstrated using PV loops. Subpulmonic RV PV loops typically lack the isovolumic contraction and relaxation phases; RV peak pressure occurs before systolic ejection, leading to a more trapezoidal RV PV loop.^41^ However, the systemic RV has prominent isovolumic contraction and relaxation phases. RV peak pressure also occurs at the end of systolic ejection with a narrow, rectangular shape (Figure 4). In addition, as the RV afterload rises out of proportion to contractility, RV function becomes uncoupled from the aorta, leading to an inefficient systemic RV and greater energy expenditure to maintain adequate RV output.^42^ PV loops in this population could in the future help develop noninvasive correlates of PVL parameters, predict adverse cardiac outcomes, and guide therapeutic interventions.

### PV loop applications in pediatric pulmonary arterial hypertension

The RV’s response to increased afterload is a key determinant of prognosis in patients with pulmonary arterial hypertension (PAH).^43^, ^44^, ^45^ This response is best investigated using the RV PV relationship and by assessing the VA coupling.^29^^,^^46^^,^^47^ Clinical studies of RV PV relationships in children with PAH are limited. Cardiac catheterization is considered higher risk in this patient population, and the focus has been on finding noninvasive surrogates to predict clinical events.^3^^,^^4^^,^^48^, ^49^, ^50^, ^51^, ^52^, ^53^, ^54^, ^55^, ^56^, ^57^ Di Maria et al^56^ first showed that the calculation of RV SW using nonsynchronous measurement of pressure (using conventional fluid-filled catheters) and SV (using thermodilution or the Fick equation) was positively correlated with functional class, the need for a septostomy, and mortality in children with PAH. Yang et al,^57^ using sequential CMR and cardiac catheterization, were able to construct RV PV loops using a computational model. They were able to demonstrate that RV SW corrected for RV EF increased over time in patients who died, underwent or were on the waiting list for lung transplantation, or experienced hemodynamic decompensation despite receiving maximal PAH-targeted therapy. The study highlighted the importance of assessing the RV SW in lieu of the position and shape of the PV loop on the PV plot. Two patients may have the same SW secondary to different combinations of pressure and volume or may have the same SV with different RV volumes and EFs. As a result, evaluating the RV’s function is best understood in relation to its load. The same concept was illustrated in another study where RV SW was interpreted in relation to the patient’s afterload. Children with low RV SW and high PVR had more adverse clinical events, such as syncope, than patients where the RV SW was matched to the PVR.^55^ It was also shown that certain markers of RV function, eg, tricuspid annular plane systolic excursion, follow a similar trajectory as the RV SW in PAH and, therefore, have the potential for being a noninvasive surrogate of RV SW.^55^ The use of noninvasive biomarkers as surrogates for the diastolic RV PV relationship has also been studied in children with PAH.^54^ Increased RV stiffness correlates with the degree of increase in RV afterload^58^^,^^59^ and VA uncoupling^60^ and is a determinant of prognosis in children with PAH.^61^

Future studies in children with PAH should focus on the validation of the noninvasive surrogates of the RV PV relationship and on comparing the different modalities currently used to obtain these surrogates. Studying the PV relationship will help us understand the differences in the RV response in the different etiologies of PAH^62^^,^^63^ and the effect of PAH-targeted medications on RV contractility, VA coupling, and prognosis.^64^, ^65^, ^66^, ^67^

### PV loop applications to guide interventions in CHD

PV loops have been used in adult structural interventions to get immediate insights into the effects of interventions such as mitral repair and transcatheter aortic valve replacement.^68^^,^^69^ Despite the advent of widespread transcatheter pulmonary valve replacement, the focus so far has been on normalization of RV volumes and functional status, the acute and long-term effects of the intervention on RV mechanics and efficiency are poorly described. RV PV loops have been used to document restrictive physiology, diastolic RV dysfunction, and abnormal lusitropy in children with repaired tetralogy of Fallot (TOF), who often have free pulmonary insufficiency and RV dilatation^70^; Gusseva et al^71^ created PV loops out of 20 repaired TOF patients prior to and after percutaneous PVR using sequential CMR and cardiac catheterization and patient-specific biomechanical models. They identified residual RV outflow obstruction as imposing greater ventricular work than isolated pulmonary regurgitation post PVR and highlighted the importance of reducing it. RV PV loops in a larger study population may help us better understand the effects of pulmonary valve replacement on right heart function and long-term outcomes.^71^^,^^72^

**Figure 5:**
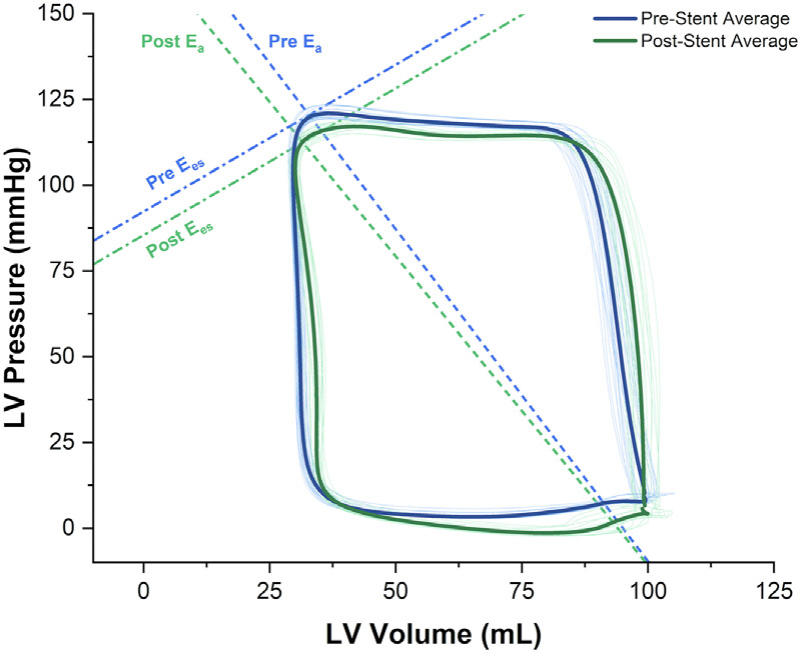
**Representative PV loops in a pediatric patient with coarctation of the aorta before and after transcatheter stent angioplasty obtained using conductance catheter positioned in the LV apex**. Blue loops: Pre-stenting, Green: After stenting. Arterial elastance (E_a_) decreased from 1.72 to 1.59 (8%) and VA coupling improved by 5%. E_es_, end-systolic elastance; PV, pressure-volume; VA, ventricular-arterial.

Transcatheter interventions for stenotic lesions in CHD have traditionally focused on angiographic improvements in vessel diameter and relief of pressure gradients, with little focus on the effects of interventions and their timing on preserving myocardial performance. For example, in a recent study, transcatheter intervention in adults that successfully relieved mild coarctation pressure gradients did not translate to decreased myocardial strain as measured using computational fluid dynamics.^73^ Furthermore, even after successful transcatheter aortic coarctation intervention in the short term, myocardial mechanics improved but did not normalize.^74^ PV loops provide an accurate means to assess the effects of repaired and unrepaired left heart obstructive lesions (examples include coarctation, aortic stenosis, and aortic arch hypoplasia) and transcatheter interventions on ventricular performance (Figure 5). These findings may aid in determining the best indications, timing, and approach for primary or follow-up intervention in these children. Several research studies on these topics are currently underway in the congenital population (eg, NCT05362721, PV Loop and Coarctation Study) through this working group and will shed more light on this in the coming years.

### PV loop applications in the diagnosis and management of patients with HF

Patients with HF have typically been categorized into those with preserved (ie, HFpEF) and reduced EFs (ie, HFrEF). PV loops in HFpEF often demonstrate an equivalent increase in E_a_ and E_es_ (ie, the E_a_/E_es_ ratio remains stable) at rest, but an elevated EDPVR and decreased SV can be unmasked with exercise.^1^^,^^75^ In addition, EDPVR and ESP also increase, coupled with a lower SV.^76^ PV loops can reveal the presence of HFpEF in some patients who have been missed by traditional echocardiographic imaging. It is well documented that noninvasive diagnostic criteria for diastolic dysfunction fail to identify some children with HFpEF.^77^ Penicka et al^78^ sought to investigate the utility of PV loop assessment in 30 outpatient adults with unexplained dyspnea (New York Heart Association classes II or III) and with normal EF. They found that 20 (67%) had evidence of HFpEF, although only 5 of these 20 met criteria for HFpEF based on noninvasive findings. It is increasingly apparent that the RV manifests diastolic dysfunction in HFpEF that cannot be explained just by increased RV afterload. Rommel et al^75^ used invasive RV and LV PV loops in 24 HFpEF patients and 9 patients without HF during basal conditions and with exercise. Although RV relaxation and cardiac output were similar at baseline, HFpEF patients demonstrated a blunted increase in cardiac output under exercise associated with prolonged RV relaxation, a decrease in SV, higher RV-filling pressures, and a marked increase in the EDPVR, highlighting the presence of RV diastolic dysfunction in compensated stages of the HFpEF syndrome. Invasive exercise hemodynamics have gained popularity and have permitted a better understanding of hemodynamics in HF patients, which is increasingly being incorporated into guidelines.^79^ Cornwell et al^80^^,^^81^ used invasive exercise PV loops in adults with continuous-flow left ventricular assist devices to demonstrate limited RV contractile reserve, marked elevations in pulmonary, left-sided filling, and right-sided filling pressures during exercise, and severe ventilatory inefficiency, which may explain the persistent impairments in their functional capacity. Levine et al^10^^,^^80^^,^^82^ combined invasive hemodynamics with echo to generate the diastolic limbs of the PV curve and have been applied to a host of populations, including healthy athletes, adults with HFpEF, and sedentary individuals. Interestingly, in a study of 46 patients with LV hypertrophy and stage B HF with preserved EF, one year of committed exercise training reversed abnormal left ventricular myocardial stiffness.^83^

Typical PV loop findings in HFrEF include a rightward shift of both EDPVR and ESPVR.^1^ In addition, there is often a higher E_a_/E_es_ ratio (typically >1.2) related to ventricular-vascular mismatch that persists with exercise. Improving atrial compliance by fenestrating the arial septum is becoming more popular in the management of the diastolic failure of the right (PAH) or left (HFpEF) heart. The study of diastology with PV loops will enhance our understanding and improve the determination of the indications and fenestration sizing in CHD-related HF.

Children undergoing heart transplantation are at risk of developing diastolic dysfunction related to factors such as graft rejection, the donor heart’s ischemic time during the transplant process, and recipient-donor size mismatch. Chowdhury et al^5^ studied 18 children post heart transplantation and found that 50% had high LV stiffness, defined as a stiffness constant, β > 0.015 ml^-1^. Those patients tended to be younger and had a lower body surface area compared to patients with normal LV stiffness. There were no significant differences in blood pressure, mixed venous oxygen saturation, EDP, E_es_, or the E_a_/E_es_ ratio, highlighting the difficulty in identifying these patients using conventional hemodynamic evaluations. The longer graft ischemic time may be the cause and has been correlated with worse ventricular stiffness.^84^ A greater understanding of the normal ranges for the EDPVR in heart transplants is needed.

### PV loop applications in pacemaker optimization

Cardiac resynchronization therapy (CRT) is an established treatment in patients with HF and dyssynchrony.^85^^,^^86^ However, the rate of nonresponse is considerable (30%-40%). Unfortunately, device settings to optimize CRT performance are not easily discernible from echocardiography, electrocardiography (ECG), or right heart catheterization. Demonstrating response to CRT can be particularly challenging in patients with CHD since echocardiographically derived estimates of ventricular function such as EF can be inaccurate or impossible to calculate in various CHDs with unusual ventricular morphologies. ECG indices are often unusable in patients with CHD since such patients often have atypical ECG patterns (commonly RBBB). The reported nonresponder definitions and rates for CRT in patients with CHD are highly variable. Noninvasive estimation of VA coupling has proven helpful in the prognostication of HF subjects and has been studied in CRT in adults with HF. In chronic HF, a combination of depressed systolic function (low ventricular elastance, E_es_) coupled to a high arterial elastance (E_a_) will result in an afterload mismatch and severely elevated E_a_/E_es_ (VA uncoupling). Stassen et al^87^ discovered that the baseline right ventricular to PA coupling, measured noninvasively using the tricuspid annular plane systolic excursion/PA systolic pressure ratio, is associated with long-term outcomes in CRT recipients. Acute changes to noninvasively measured VA coupling with CRT did not correlate with the 3-year prognosis in a recent study of adults with HF.^88^

Invasive PV loop analysis provides a way to demonstrate the acute hemodynamic effects of CRT and thereby instantly visualize the impact of different lead locations and pacing strategies on ventricular performance. This has been successfully demonstrated in several studies in adult HF.^89^, ^90^, ^91^, ^92^, ^93^ Protocols describing their use in adults with HF have been published^89^ where baseline PV loops are compared with PV loops recorded at different CRT settings. The invasive PV loop indices that are frequently described for CRT optimization include SW and dP/dt max (maximal rate of left ventricular pressure rise). The lead location or pacing strategy that provides the highest positive change in SW, or dP/dt, from the baseline condition is chosen to be the optimal setting for that patient. The acute hemodynamic impact of CRT can thus be readily observed and optimized. Invasive PV loops have confirmed acute enhancements in VA coupling during CRT as a result of beneficial changes in both determinants (ie, E_es_ increase and E_a_ reduction) secondary to effects of sympathetic activity.^94^ Zweerink et al^89^ compared dP/dt max optimization and SW optimization in 41 adult HF patients who underwent CRT and invasive PV loops. They showed that PV loop-guided optimization of CRT resulted in an approximately one-third improvement in SW compared to conventional CRT. Comparing both optimization strategies, dP/dt max favored improved contractility, whereas SW optimization improved VA coupling (45% vs 32%; *P* < .001). They further showed that SW optimization was more predictive of long-term response to CRT than dP/dt max.

**Figure 6:**
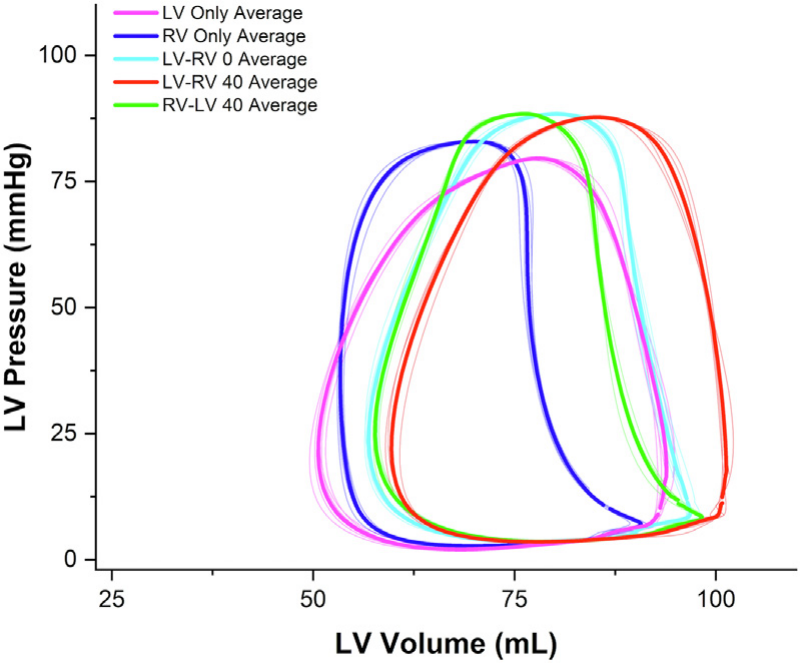
**Representative PV loops obtained using a conductance catheter in a patient with congenitally corrected transposition of great arteries status post double switch operation during CRT**. Settings are described based on which ventricle was paced first followed by the ventriculo-ventricular delay. Purple: LV only. Blue: RV only. Turquoise: LV-RV 0 msec. Red: LV-RV 40 msec. Green: RV-LV 40 msec. LV-RV 40 msec provided the highest LV stroke work. Compared to this setting, RV only pacing led to a 30% decline in LV stroke work. CRT, cardiac resynchronization therapy; LV, left ventricle; PV, pressure-volume; RV, right ventricle.

Literature on the use of invasive PV loops for CRT optimization in CHD is lacking. Karpawich et al^95^ demonstrated the use of invasive dP/dt max measured using conventional fluid-filled catheters to guide patient selection for CRT placement among 40 CHD patients with HF. An arbitrary cutoff of improved dP/dt max of at least 15% over baseline values was chosen to show a potentially effective CRT response, and those patients (26/40, 65%) were offered CRT. Based on long term and stable improvements in New York Heart Association functional class and invasive dP/dt max, they concluded that short-term paced contractility response testing could identify those CHD patients who are likely to respond to CRT regardless of anatomy. Figure 6 depicts real-life invasive PV loops demonstrating the acute hemodynamic impact of CRT in a patient with congenitally corrected great artery transposition after a double switch repair (Mustard baffle and arterial switch operation). As seen clearly, pacer settings in which the RV is paced ahead of the LV led to significant declines in the LV SW. The optimal settings based on SW optimization for this patient were found to be CRT with the LV activation 40 ms ahead of the RV activation.

## Limitations

Despite its ability to provide a valuable assessment of myocardial performance and cardiac hemodynamics, invasive PV loop assessment has several limitations. The primary limitation is the invasive nature, which makes serial follow-up measurements to help guide management challenging. The continued development of reliable and reproducible noninvasive measures as described above, particularly using CMR, holds great promise in this regard. The techniques for obtaining high-quality loops, especially in small children, and accurate calibration for analysis and interpretation still involve certain assumptions or subjective decisions, hence the need for standardization of techniques and reporting practices. Lastly, the cost of the equipment and catheters remains a challenge until reimbursement is streamlined.

## Conclusion and future directions

PV loop analysis provides a powerful tool to assess the complex ventricular mechanics in detail. The resurgence of invasive PV loop in conjunction with modeled PV loops from advanced imaging and ICMR has the potential to greatly improve our understanding of the pathophysiology of various CHDs and may shape the interventions that we perform. There is a crucial need to develop normal values and references for PV loop parameters in children. The large variety of CHDs, small volume studies, and limited centers with PV loop infrastructure are some of the barriers. Future efforts should focus on identifying predictors of outcomes and optimal cutoffs for interventions using PV loops with the goal of optimizing long term ventricular performance. Greater engagement among various researchers in the form of multicenter collaborative projects and a national registry may be crucial in this regard.

## References

[1] Bastos M.B., Burkhoff D., Maly J. (2020). Invasive left ventricle pressure-volume analysis: overview and practical clinical implications. Eur Heart J.

[2] Brener M.I., Masoumi A., Ng V.G. (2022). Invasive right ventricular pressure-volume analysis: basic principles, clinical applications, and practical recommendations. Circ Heart Fail.

[3] Chowdhury S.M., Butts R.J., Taylor C.L. (2016). Validation of noninvasive measures of left ventricular mechanics in children: a simultaneous echocardiographic and conductance catheterization study. J Am Soc Echocardiogr.

[4] Chowdhury S.M., Butts R.J., Taylor C.L. (2018). Longitudinal measures of deformation are associated with a composite measure of contractility derived from pressure-volume loop analysis in children. Eur Heart J Cardiovasc Imaging.

[5] Chowdhury S.M., Butts R.J., Hlavacek A.M. (2018). Echocardiographic detection of increased ventricular diastolic stiffness in pediatric heart transplant recipients: a pilot study. J Am Soc Echocardiogr.

[6] Butts R.J., Chowdhury S.M., Buckley J. (2015). Comparison of echocardiographic and pressure-volume loop indices of systolic function in patients with single ventricle physiology: a preliminary report. Congenit Heart Dis.

[7] Butts R.J., Hsia T.Y., Hamilton Baker G., MOCHA Investigators (2013). Feasibility of conductance catheter-derived pressure-volume loops to investigate ventricular mechanics in shunted single ventricles. Cardiol Young.

[8] Klotz S., Hay I., Dickstein M.L. (2006). Single-beat estimation of end-diastolic pressure-volume relationship: a novel method with potential for noninvasive application. Am J Physiol Heart Circ Physiol.

[9] Burkhoff D., Mirsky I., Suga H. (2005). Assessment of systolic and diastolic ventricular properties via pressure-volume analysis: a guide for clinical, translational, and basic researchers. Am J Physiol Heart Circ Physiol.

[10] Hieda M., Howden E., Shibata S. (2018). Impact of lifelong exercise training dose on ventricular-arterial coupling. Circulation.

[11] Alfakih K., Plein S., Thiele H., Jones T., Ridgway J.P., Sivananthan M.U. (2003). Normal human left and right ventricular dimensions for MRI as assessed by turbo gradient echo and steady-state free precession imaging sequences. J Magn Reson Imaging.

[12] Brener M.I., Burkhoff D., Sunagawa K. (2020). Effective arterial elastance in the pulmonary arterial circulation: derivation, assumptions, and clinical applications. Circ Heart Fail.

[13] Kass D.A., Midei M., Graves W., Brinker J.A., Maughan W.L. (1988). Use of a conductance (volume) catheter and transient inferior vena caval occlusion for rapid determination of pressure-volume relationships in man. Cathet Cardiovasc Diagn.

[14] Burkhoff D. (1990). The conductance method of left ventricular volume estimation. Methodologic limitations put into perspective. Circulation.

[15] Burkhoff D. (2013). Pressure-volume loops in clinical research: a contemporary view. J Am Coll Cardiol.

[16] Baan J., van der Velde E.T., de Bruin H.G. (1984). Continuous measurement of left ventricular volume in animals and humans by conductance catheter. Circulation.

[17] Kornet L., Schreuder J.J., van der Velde E.T., Jansen J.R. (2001). The volume-dependency of parallel conductance throughout the cardiac cycle and its consequence for volume estimation of the left ventricle in patients. Cardiovasc Res.

[18] Steendijk P., Staal E., Jukema J.W., Baan J. (2001). Hypertonic saline method accurately determines parallel conductance for dual-field conductance catheter. Am J Physiol Heart Circ Physiol.

[19] Looga R. (2005). The Valsalva manoeuvre--cardiovascular effects and performance technique: a critical review. Respir Physiol Neurobiol.

[20] Ten Brinke E.A., Burkhoff D., Klautz R.J. (2010). Single-beat estimation of the left ventricular end-diastolic pressure-volume relationship in patients with heart failure. Heart.

[21] Steflik D., Butts R.J., Baker G.H. (2017). A preliminary comparison of two-dimensional speckle tracking echocardiography and pressure-volume loop analysis in patients with Fontan physiology: the role of ventricular morphology. Echocardiography.

[22] Veeram Reddy S.R., Arar Y., Zahr R.A. (2020). Invasive cardiovascular magnetic resonance (iCMR) for diagnostic right and left heart catheterization using an MR-conditional guidewire and passive visualization in congenital heart disease. J Cardiovasc Magn Reson.

[23] Soldo S.J., Norris S.L., Gober J.R., Haywood L.J., Colletti P.M., Terk M. (1994). MRI-derived ventricular volume curves for the assessment of left ventricular function. Magn Reson Imaging.

[24] La Gerche A., Claessen G., Van de Bruaene A. (2013). Cardiac MRI: a new gold standard for ventricular volume quantification during high-intensity exercise. Circ Cardiovasc Imaging.

[25] Castellanos D.A., Škardová K., Bhattaru A. (2021). Left ventricular torsion obtained using equilibrated warping in patients with repaired tetralogy of Fallot. Pediatr Cardiol.

[26] Wong J., Pushparajah K., de Vecchi A. (2017). Pressure-volume loop-derived cardiac indices during dobutamine stress: a step towards understanding limitations in cardiac output in children with hypoplastic left heart syndrome. Int J Cardiol.

[27] Gusseva M., Castellanos D.A., Greer J.S. (2023). Time-synchronization of interventional cardiovascular magnetic resonance data using a biomechanical model for pressure-volume loop analysis. J Magn Reson Imaging.

[28] Wang V.Y., Lam H.I., Ennis D.B., Cowan B.R., Young A.A., Nash M.P. (2009). Modelling passive diastolic mechanics with quantitative MRI of cardiac structure and function. Med Image Anal.

[29] Redington A.N., Rigby M.L., Shinebourne E.A., Oldershaw P.J. (1990). Changes in the pressure-volume relation of the right ventricle when its loading conditions are modified. Br Heart J.

[30] Schlangen J., Fischer G., Petko C. (2013). Arterial elastance and its impact on intrinsic right ventricular function in palliated hypoplastic left heart syndrome. Int J Cardiol.

[31] Caravita S., Baratto C. (2023). Understanding mechanisms of Fontan failure: exercise haemodynamics to unmask diastolic dysfunction, again. Eur J Heart Fail.

[32] Egbe A.C., Miranda W.R., Anderson J.H., Borlaug B.A. (2020). Hemodynamic and clinical implications of impaired pulmonary vascular reserve in the Fontan circulation. J Am Coll Cardiol.

[33] Miranda W.R., Borlaug B.A., Jain C.C. (2023). Exercise-induced changes in pulmonary artery wedge pressure in adults post-Fontan versus heart failure with preserved ejection fraction and non-cardiac dyspnoea. Eur J Heart Fail.

[34] Budts W., Ravekes W.J., Danford D.A., Kutty S. (2020). Diastolic heart failure in patients with the Fontan circulation: a review. JAMA Cardiol.

[35] Brida M., Diller G.P., Gatzoulis M.A. (2018). Systemic right ventricle in adults with congenital heart disease: anatomic and phenotypic spectrum and current approach to management. Circulation.

[36] Konstam M.A., Kiernan M.S., Bernstein D. (2018). Evaluation and management of right-sided heart failure: a scientific statement from the American Heart Association. Circulation.

[37] Stout K.K., Daniels C.J., Aboulhosn J.A. (2019). 2018 AHA/ACC guideline for the management of adults with congenital heart disease: executive summary: a report of the American College of Cardiology/American Heart Association Task Force on Clinical Practice Guidelines. J Am Coll Cardiol.

[38] Sanchez-Quintana D., Climent V., Ho S.Y., Anderson R.H. (1999). Myoarchitecture and connective tissue in hearts with tricuspid atresia. Heart.

[39] Sedmera D. (2005). Form follows function: developmental and physiological view on ventricular myocardial architecture. Eur J Cardiothorac Surg.

[40] Lahm T., Douglas I.S., Archer S.L. (2018). Assessment of right ventricular function in the research setting: knowledge gaps and pathways forward. An official American Thoracic Society research statement. Am J Respir Crit Care Med.

[41] Friedberg M.K., Redington A.N. (2014). Right versus left ventricular failure: differences, similarities, and interactions. Circulation.

[42] Wauthy P., Naeije R., Brimioulle S. (2005). Left and right ventriculo-arterial coupling in a patient with congenitally corrected transposition. Cardiol Young.

[43] Abman S.H., Hansmann G., Archer S.L. (2015). Pediatric pulmonary hypertension: guidelines from the American Heart Association and American Thoracic Society. Circulation.

[44] Rosenzweig E.B., Abman S.H., Adatia I. (2019). Paediatric pulmonary arterial hypertension: updates on definition, classification, diagnostics and management. Eur Respir J.

[45] Hansmann G., Koestenberger M., Alastalo T.P. (2019). 2019 updated consensus statement on the diagnosis and treatment of pediatric pulmonary hypertension: the European Pediatric Pulmonary Vascular Disease Network (EPPVDN), endorsed by AEPC, ESPR and ISHLT. J Heart Lung Transplant.

[46] Gaynor S.L., Maniar H.S., Bloch J.B., Steendijk P., Moon M.R. (2005). Right atrial and ventricular adaptation to chronic right ventricular pressure overload. Circulation.

[47] Hsu S., Simpson C.E., Houston B.A. (2020). Multi-beat right ventricular-arterial coupling predicts clinical worsening in pulmonary arterial hypertension. J Am Heart Assoc.

[48] Tello K., Dalmer A., Vanderpool R. (2019). Cardiac magnetic resonance imaging-based right ventricular strain analysis for assessment of coupling and diastolic function in pulmonary hypertension. JACC Cardiovasc Imaging.

[49] Tello K., Wan J., Dalmer A. (2019). Validation of the tricuspid annular plane systolic excursion/systolic pulmonary artery pressure ratio for the assessment of right ventricular-arterial coupling in severe pulmonary hypertension. Circ Cardiovasc Imaging.

[50] Tello K., Dalmer A., Vanderpool R. (2020). Right ventricular function correlates of right atrial strain in pulmonary hypertension: a combined cardiac magnetic resonance and conductance catheter study. Am J Physiol Heart Circ Physiol.

[51] Breeman K.T.N., Dufva M., Ploegstra M.J. (2019). Right ventricular-vascular coupling ratio in pediatric pulmonary arterial hypertension: a comparison between cardiac magnetic resonance and right heart catheterization measurements. Int J Cardiol.

[52] Levy P.T., El Khuffash A., Woo K.V., Hauck A., Hamvas A., Singh G.K. (2019). A novel noninvasive index to characterize right ventricle pulmonary arterial vascular coupling in children. JACC Cardiovasc Imaging.

[53] Sanz J., García-Alvarez A., Fernández-Friera L. (2012). Right ventriculo-arterial coupling in pulmonary hypertension: a magnetic resonance study. Heart.

[54] Okumura K., Slorach C., Mroczek D. (2014). Right ventricular diastolic performance in children with pulmonary arterial hypertension associated with congenital heart disease: correlation of echocardiographic parameters with invasive reference standards by high-fidelity micromanometer catheter. Circ Cardiovasc Imaging.

[55] Di Maria M.V., Campbell K.R., Burkett D.A. (2020). Parameters of right ventricular function reveal ventricular-vascular mismatch as determined by right ventricular stroke work versus pulmonary vascular resistance in children with pulmonary hypertension. J Am Soc Echocardiogr.

[56] Di Maria M.V., Younoszai A.K., Mertens L. (2014). RV stroke work in children with pulmonary arterial hypertension: estimation based on invasive haemodynamic assessment and correlation with outcomes. Heart.

[57] Yang W., Marsden A.L., Ogawa M.T. (2018). Right ventricular stroke work correlates with outcomes in pediatric pulmonary arterial hypertension. Pulm Circ.

[58] Rain S., Handoko M.L., Trip P. (2013). Right ventricular diastolic impairment in patients with pulmonary arterial hypertension. Circulation.

[59] Rain S., Andersen S., Najafi A. (2016). Right ventricular myocardial stiffness in experimental pulmonary arterial hypertension: relative contribution of fibrosis and myofibril stiffness. Circ Heart Fail.

[60] Alaa M., Abdellatif M., Tavares-Silva M. (2016). Right ventricular end-diastolic stiffness heralds right ventricular failure in monocrotaline-induced pulmonary hypertension. Am J Physiol Heart Circ Physiol.

[61] Takatsuki S., Nakayama T., Jone P.N. (2012). Tissue Doppler imaging predicts adverse outcome in children with idiopathic pulmonary arterial hypertension. J Pediatr.

[62] Tedford R.J., Mudd J.O., Girgis R.E. (2013). Right ventricular dysfunction in systemic sclerosis-associated pulmonary arterial hypertension. Circ Heart Fail.

[63] Hsu S., Kokkonen-Simon K.M., Kirk J.A. (2018). Right ventricular myofilament functional differences in humans with systemic sclerosis-associated versus idiopathic pulmonary arterial hypertension. Circulation.

[64] Rex S., Missant C., Claus P., Buhre W., Wouters P.F. (2008). Effects of inhaled iloprost on right ventricular contractility, right ventriculo-vascular coupling and ventricular interdependence: a randomized placebo-controlled trial in an experimental model of acute pulmonary hypertension. Crit Care.

[65] Vanderpool R.R., Desai A.A., Knapp S.M. (2017). How prostacyclin therapy improves right ventricular function in pulmonary arterial hypertension. Eur Respir J.

[66] Friedman S.H., Tedford R.J. (2022). Are you coupled? Hemodynamic phenotyping in pulmonary hypertension. Function (Oxf).

[67] Ghio S., Crimi G., Houston B. (2021). Nonresponse to acute vasodilator challenge and prognosis in heart failure with pulmonary hypertension. J Card Fail.

[68] Brener M.I., Burkhoff D., Sarraf M. (2021). Right ventricular pressure-volume analysis before and after transcatheter leaflet approximation for severe mitral regurgitation. JAMA Cardiol.

[69] Sarraf M., Burkhoff D., Brener M.I. (2021). First-in-man 4-chamber pressure-volume analysis during transcatheter aortic valve replacement for bicuspid aortic valve disease. JACC Case Rep.

[70] Apitz C., Latus H., Binder W. (2010). Impact of restrictive physiology on intrinsic diastolic right ventricular function and lusitropy in children and adolescents after repair of tetralogy of Fallot. Heart.

[71] Gusseva M., Hussain T., Friesen C.H. (2021). Biomechanical modeling to inform pulmonary valve replacement in tetralogy of Fallot patients after complete repair. Can J Cardiol.

[72] Gusseva M., Hussain T., Hancock Friesen C., Greil G., Chapelle D., Chabiniok R. (2021). Prediction of ventricular mechanics after pulmonary valve replacement in tetralogy of Fallot by biomechanical modeling: a step towards precision healthcare. Ann Biomed Eng.

[73] Keshavarz-Motamed Z., Rikhtegar Nezami F., Partida R.A. (2016). Elimination of transcoarctation pressure gradients has no impact on left ventricular function or aortic shear stress after intervention in patients with mild coarctation. JACC Cardiovasc Interv.

[74] Kheiwa A., Aggarwal S., Forbes T.J., Turner D.R., Kobayashi D. (2016). Impact of transcatheter intervention on myocardial deformation in patients with coarctation of the aorta. Pediatr Cardiol.

[75] Rommel K.P., von Roeder M., Oberueck C. (2018). Load-independent systolic and diastolic right ventricular function in heart failure with preserved ejection fraction as assessed by resting and handgrip exercise pressure-volume loops. Circ Heart Fail.

[76] Kawaguchi M., Hay I., Fetics B., Kass D.A. (2003). Combined ventricular systolic and arterial stiffening in patients with heart failure and preserved ejection fraction: implications for systolic and diastolic reserve limitations. Circulation.

[77] Dragulescu A., Mertens L., Friedberg M.K. (2013). Interpretation of left ventricular diastolic dysfunction in children with cardiomyopathy by echocardiography: problems and limitations. Circ Cardiovasc Imaging.

[78] Penicka M., Bartunek J., Trakalova H. (2010). Heart failure with preserved ejection fraction in outpatients with unexplained dyspnea: a pressure-volume loop analysis. J Am Coll Cardiol.

[79] McDonagh T.A., Metra M., Adamo M. (2022). 2021 ESC Guidelines for the diagnosis and treatment of acute and chronic heart failure developed by the task force for the diagnosis and treatment of acute and chronic heart failure of the European Society of Cardiology (ESC) with the special contribution of the Heart Failure Association (HFA) of the ESC. Eur J Heart Fail.

[80] Cornwell W.K., Tran T., Cerbin L. (2020). New insights into resting and exertional right ventricular performance in the healthy heart through real-time pressure-volume analysis. J Physiol.

[81] Tran T., Muralidhar A., Hunter K. (2021). Right ventricular function and cardiopulmonary performance among patients with heart failure supported by durable mechanical circulatory support devices. J Heart Lung Transplant.

[82] Edward J.A., Cerbin L.P., Groves D.W. (2022). Right ventricular dysfunction during endurance exercise as determined by pressure-volume analysis. JACC Case Rep.

[83] Hieda M., Sarma S., Hearon C.M. (2021). One-year committed exercise training reverses abnormal left ventricular myocardial stiffness in patients with stage B heart failure with preserved ejection fraction. Circulation.

[84] Schroeder L.W., Chowdhury S.M., Burnette A.L. (2018). Longer ischemic time is associated with increased ventricular stiffness as measured by pressure-volume loop analysis in pediatric heart transplant recipients. Pediatr Cardiol.

[85] Cleland J.G., Daubert J.C., Erdmann E. (2005). The effect of cardiac resynchronization on morbidity and mortality in heart failure. N Engl J Med.

[86] Moss A.J., Hall W.J., Cannom D.S. (2009). Cardiac-resynchronization therapy for the prevention of heart-failure events. N Engl J Med.

[87] Stassen J., Galloo X., Hirasawa K. (2022). Right ventricular–pulmonary artery coupling in cardiac resynchronization therapy: evolution and prognosis. ESC Heart Fail.

[88] De Vecchi F., Prenna E., Boggio E. (2017). Prognostic role of ventricular-arterial coupling in patients with left ventricular dysfunction submitted to cardiac resynchronization therapy. J Am Coll Cardiol.

[89] Zweerink A., Salden O.A.E., van Everdingen W.M. (2019). Hemodynamic optimization in cardiac resynchronization therapy: should we aim for dP/dtmax or stroke work?. JACC Clin Electrophysiol.

[90] van Everdingen W.M., Zweerink A., Salden O.A.E. (2018). Pressure-volume loop analysis of multipoint pacing with a quadripolar left ventricular lead in cardiac resynchronization therapy. JACC Clin Electrophysiol.

[91] Steendijk P., Tulner S.A., Bax J.J. (2006). Hemodynamic effects of long-term cardiac resynchronization therapy: analysis by pressure-volume loops. Circulation.

[92] de Roest G.J., Allaart C.P., de Haan S. (2011). Effects of QRS duration and pacing location on pressure-volume loop evaluation of cardiac resynchronization therapy in end-stage heart failure. Am J Cardiol.

[93] de Roest G.J., Allaart C.P., Kleijn S.A. (2013). Prediction of long-term outcome of cardiac resynchronization therapy by acute pressure-volume loop measurements. Eur J Heart Fail.

[94] Pieragnoli P., Perego G.B., Ricciardi G. (2015). Cardiac resynchronization therapy acutely improves ventricular-arterial coupling by reducing the arterial load: assessment by pressure-volume loops. Pacing Clin Electrophysiol.

[95] Karpawich P.P., Bansal N., Samuel S., Sanil Y., Zelin K. (2017). 16 years of cardiac resynchronization pacing among congenital heart disease patients: direct contractility (dP/dt-max) screening when the guidelines do not apply. JACC Clin Electrophysiol.

